# Integrated Transcriptomic Analysis Reveals Reciprocal Interactions between SARS-CoV-2 Infection and Multi-Organ Dysfunction, Especially the Correlation of Renal Failure and COVID-19

**DOI:** 10.3390/life14080960

**Published:** 2024-07-30

**Authors:** Pai Li, Meng Liu, Wei-Ming He

**Affiliations:** 1 Capricorn Partner, 3000 Leuven, Belgium; 2 Lee Kong Chian School of Medicine, Nanyang Technological University, Singapore 308232, Singapore; 3 School of Chemical Biology and Biotechnology, Peking University Shenzhen Graduate School, Shenzhen 518055, China

**Keywords:** COVID-19, inflammation, renal failure, transcriptomic analysis, organ dysfunction

## Abstract

The COVID-19 pandemic, which is caused by the SARS-CoV-2 virus, has resulted in extensive health challenges globally. While SARS-CoV-2 primarily targets the respiratory system, clinical studies have revealed that it could also affect multiple organs, including the heart, kidneys, liver, and brain, leading to severe complications. To unravel the intricate molecular interactions between the virus and host tissues, we performed an integrated transcriptomic analysis to investigate the effects of SARS-CoV-2 on various organs, with a particular focus on the relationship between renal failure and COVID-19. A comparative analysis showed that SARS-CoV-2 triggers a systemic immune response in the brain, heart, and kidney tissues, characterized by significant upregulation of cytokine and chemokine secretion, along with enhanced migration of lymphocytes and leukocytes. A weighted gene co-expression network analysis demonstrated that SARS-CoV-2 could also induce tissue-specific transcriptional profiling. More importantly, single-cell sequencing revealed that COVID-19 patients with renal failure exhibited lower metabolic activity in lung epithelial and B cells, with reduced ligand–receptor interactions, especially CD226 and ICAM, suggesting a compromised immune response. A trajectory analysis revealed that COVID-19 patients with renal failure exhibited less mature alveolar type 1 cells. Furthermore, these patients showed potential fibrosis in the hearts, liver, and lung increased extracellular matrix remodeling activities. However, there was no significant metabolic dysregulation in the liver of COVID-19 patients with renal failure. Candidate drugs prediction by Drug Signatures database and LINCS L1000 Antibody Perturbations Database underscored the importance of considering multi-organ effects in COVID-19 management and highlight potential therapeutic strategies, including targeting viral entry and replication, controlling tissue fibrosis, and alleviating inflammation.

## 1. Introduction

In 2019, a new type of coronavirus associated with respiratory infections and pneumonia was initially identified in Wuhan, China, prompting widespread global concern. Designated as severe acute respiratory syndrome coronavirus 2 (SARS-CoV-2), it is a positive-stranded single-stranded RNA virus [1,2,3]. SARS-CoV-2 can infiltrate the human body through various entry points, including the upper respiratory tract, eyes, and nasal or oral cavity, where the angiotensin-converting enzyme 2 (ACE2) receptor is expressed on the surface of these tissues [4,5].

The predominant clinical manifestation in COVID-19 patients is characterized by progressive respiratory failure, which results from both alveolar damage and perivascular T cell infiltration [6,7]. However, the impacts of COVID-19 extend beyond the respiratory system to affect various organs. Clinical evaluation revealed significant implications of COVID-19 for multiple organ systems. For the cardiac system, it could lead to impaired ventricular function, deep vein thrombosis, and myocarditis [8,9,10]. In the liver, the virus can cause acute liver injury with upregulated alanine transaminase (ALT) and aspartate aminotransferase (AST) activity, alongside hepatitis-like symptoms, such as jaundice and abdominal pain. Coagulation abnormalities increase the risk of complications like hepatic vein thrombosis, necessitating vigilant liver function monitoring [11]. COVID-19 also causes neurological symptoms in addition to systemic and respiratory ones, affecting both the central and peripheral nervous systems [12]. Furthermore, COVID-19 can potentially affect the adrenal glands and gonads, impacting steroidogenesis and fertility [13]. The systemic inflammation and direct cellular effects induced by the virus have the potential to disrupt normal endocrine function. This disruption may manifest as conditions like pancytopenia, characterized by a decrease in all blood cell types, leading to symptoms such as fatigue, increased infection susceptibility, and bleeding tendencies [14]. Additionally, COVID-19 can trigger pancreatitis, causing symptoms like abdominal pain and digestive disturbances [15]. More importantly, organ failure can occur due to direct viral damage, systemic inflammation, and the other hyper-immune response [16,17]. Monitoring and managing these complications are crucial in the comprehensive care of COVID-19 patients.

Acute kidney injury (AKI) is a significant complication observed in COVID-19 patients [18]. The virus can directly infect renal cells, causing tubular injury and inflammation. Additionally, systemic inflammation, cytokine release syndrome, and hemodynamic instability contribute to renal dysfunction. Among individuals diagnosed with COVID-19, those with late chronic kidney disease (CKD) have a higher risk of mortality compared to individuals with normal kidney function or mild CKD [19]. The observed abnormalities imply the multi-organ impact of SARS-CoV-2 infection, offering insights into the broader pathophysiological implications of COVID-19. However, most research relies on clinical observations, and there is still insufficient understanding of the molecular mechanisms underlying the impact of COVID-19 on kidney dysfunction.

The intertwining of COVID-19 and metabolic processes has emerged as a novel area of research. COVID-19 not only induces acute respiratory distress but also elicits systemic metabolic alterations. Different organs respond differently to the virus. In the cardiovascular system, COVID-19 has been associated with metabolic disturbances, including altered lipid profiles and endothelial dysfunction. These metabolic changes could reciprocally contribute to the increased risk of cardiovascular complications, which have been observed in some patients [20,21]. In liver, the virus appears to induce systemic inflammation, potentially influencing metabolic homeostasis. Studies have reported hepatic abnormalities and altered glucose metabolism in COVID-19 patients, suggesting broader metabolic involvement [22,23]. Renal complications have also become a concern, with observations of acute kidney injury and persistent renal abnormalities post-infection. Metabolic dysregulation, including disruptions in glucose metabolism, could contribute to these renal manifestations [24]. Examining the metabolic profiles at organ- and cell-specific levels could capture the heterogeneity of cellular responses, providing more understanding of the disease.

The metabolic activities in different organs during COVID-19 infection, especially the intricate interaction between kidney failure and COVID-19, remains poorly understood. A comprehensive understanding of these influences is crucial for developing targeted therapeutic strategies and addressing the long-term health implications of COVID-19 survivors. In this study, we conducted an analysis of transcriptional profiling to explore the heterogeneity and homogeneity across different organs, which could serve as a foundational step for potential drug development.

## 2. Materials and Methods

### 2.1. Information of Transcriptomics Analysis

The aim of this study was to investigate the transcriptional features of SARS-CoV-2 infection on different organs. We downloaded the bulk RNA-seq datasets of SARS-CoV-2 infected lung, choroid plexus organoids, and adult cardiomyocytes from the GEO database of the National Center for Biotechnology Information (NCBI) (https://www.ncbi.nlm.nih.gov/geo/, accessed on 7 January 2024). For the lung dataset (GSE150316), we analyzed 5 infected lung samples, which were derived from autopsy samples of COVID-19-positive patients, and 5 uninfected lung specimens as the negative controls [25]. For the choroid plexus organoids dataset (GSE157852), we analyzed 3 iPSCs-derived choroid plexus organoids samples after 72 h SARS-CoV-2 infection and 3 mock 72 h post-infection samples [26]. For the cardiomyocytes dataset (GSE151879), we analyzed 3 adult human cardiomyocytes samples with SARS-CoV-2 infection and 3 adult human cardiomyocytes samples with mock treatment [27]. For the human embryonic kidney cell dataset (GSE189706), we analyzed 3 human embryonic kidney cells transfected with SARS-CoV-2 N proteins for 24 h and 3 transfected with GFP for 24 h as the controls [28]. To further analyze the infiltrating immune response, the single-cell RNA-seq datasets of whole blood from healthy individuals (4 samples) and COVID-19 patients (4 samples) were sourced from GSE163668 [29]. The single-cell RNA-seq datasets from kidney samples of healthy individuals (6 samples), diabetic kidney disease (DKD) patients (3 samples), and hypertension chronic kidney disease (CKD) patients (3 samples) were obtained from GSE183276 [30]. To investigate the correlation of kidney failure and COVID-19 severity, we used the single nucleus RNA-seq dataset of lung, liver, kidney, and heart tissues from 3 COVID-19-affected autopsy samples and another 3 COVID-19 patients with kidney failure (GSE171668) [31]. The detailed sample information is listed in Table S6.

### 2.2. Gene Set Enrichment Analysis

A gene set enrichment analysis (GSEA) was performed with software from https://www.gsea-msigdb.org/gsea/index.jsp, accessed on 20 January 2024 [32,33]. Expression matrices for the SARS-CoV-2 infection and control groups were used as the input. The Kyoto Encyclopedia of Genes and Genomes (KEGG) database was used as a reference for the pathway enrichment analysis [34]. The number of permutations was 1000, and the permutation type was gene set. The enrichment statistic method was weighted, and the metric for ranking genes was Signal2Noise. Pathways with a *p*-value < 0.05 and a normalized enrichment score (NES) > 1 were considered as significantly changed.

### 2.3. Weighted Gene Co-Expression Network Analysis (WGCNA)

The raw RNA-seq count data were combined across different organs and preprocessed by filtering out low-expression genes. The soft-thresholding power was determined by analyzing the scale-free topology fit index and mean connectivity. The adjacency matrix was constructed based on the power value, which was then transformed into a topological overlap matrix (TOM) to minimize noise and spurious associations. Genes were hierarchically clustered based on the TOM-based dissimilarity measure. Modules were summarized using the module eigengene, and similar modules were merged based on eigengene correlation. The resulting gene modules were further analyzed for their biological significance and association with clinical traits.

### 2.4. Protein–Protein Interaction Network

We performed a comparative analysis between the SARS-CoV-2 and control groups from each bulk RNA-seq dataset. The cut-off criteria for different expressed genes (DEGs) were based on a *p*-value < 0.01 and |Fold change| ≥ 1.5. Online Venn diagram software (https://bioinformatics.psb.ugent.be/webtools/Venn/, accessed on 20 February 2024) was used to visualize the shared DEGs among the 3 datasets. We input the DEGs to STRING (Search Tool for the Retrieval of Interacting Genes and Proteins, https://string-db.org/, accessed on 20 February 2024) for protein–protein interactions with the median confidence score > 0.4 [35]. The PPI network was visualized by Cytoscape v.3.9.0 [36].

### 2.5. Identification Hub Genes

To identify the hub genes, we used Cytohubba with Cytoscape to rank the central elements using the maximal clique centrality (MCC) method. The top 15 hub genes were selected for further analysis.

### 2.6. Gene–Disease Association and Candidate Drugs Prediction

The Disease Gene Network (DisGeNET) was used to identify the hub gene–disease relationship and reveal other related complications [37]. Gene-associated diseases with a *p*-value < 0.05 were selected, and the top 10 were selected for further analysis. We used the Drug Signatures database (DSigDB) to predict the candidate drugs with the hub genes [38]. All of the analyses were performed via Enrichr (https://maayanlab.cloud/Enrichr/, accessed on 20 February 2024) [39]. Potential drugs were selected with a *p*-value < 0.05, and we extracted the top 10 for further analysis.

### 2.7. Prediction of Candidate Antibody by LINCS L1000 Antibody Perturbations Database

The prediction of candidate antibodies was performed using SigCom LINCS (https://maayanlab.cloud/sigcom-lincs/#/SignatureSearch/UpDown, accessed on 2 July 2024) [40]. Differentially expressed genes (DEGs) from each bulk RNA-seq dataset were used as input. The LINCS L1000 Antibody Perturbations Database served as the reference. The top 10 reverser antibodies (ranking by z-score) were selected for further analysis. 

### 2.8. Single-Nucleus and Single-Cell RNA Sequencing Data Processing

All the single-nucleus and single-cell RNA sequencing datasets were processed through Seurat v4.0.0 [41]. The ambient RNA contamination was removed by SoupX v1.5.061 based on the calculated contamination fraction. The doublets and low-quality cells were removed based on annotated metadata. The gene expression counts were normalized, scaled, and then clustered with uniform manifold approximation and projection (UMAP) space. Individual clusters were annotated based on the expression of lineage-specific markers and annotated metadata. 

### 2.9. Single-Cell Metabolic Pathway Enrichment Calculation

The gene set enrichment analysis was performed with R package VISION based on metabolic pathways from the KEGG database [42]. Firstly, the gene expression data were normalized to ensure comparability across the samples, and log transformation was used to stabilize the variance. Gene set variation analysis (GSVA) was further used to calculate the non-parametric and unsupervised results. To account for multiple comparisons, the Benjamini–Hochberg procedure was used to control the false discovery rate (FDR).

### 2.10. Ligand-Receptor Analysis on Single-Cell Level

Cell–cell communication analysis was conducted using CellChat (v1.1.3 R), which includes the identification of ligand–receptor interactions between cell types [43]. The CellChat objects for COVID-19 with/without kidney failure were generated using the createCellChat function. The objects were subsequently preprocessed using the identifyOverExpressedInteractions and projectData functions. Following this, the communication probability was computed using the computeCommunProb function with the parameters type = “truncatedMean” and trim = 0.001.

### 2.11. Subcluster and Trajectory Analysis

Lung epithelial cells, hepatocytes, and cardiomyocytes were isolated with cell type annotation. Differentially expressed genes (DEGs) were calculated with the FindMarkers function with |log2FC| > 0.25 and *p*-value < 0.01. The DEGs were enriched with the GO Biological Process in DAVID Bioinformatics Resources [44,45]. The proliferation activity was calculated with the CellCycleScoring function in Seurat. A trajectory analysis was performed using Monocle 3 for inferring developmental trajectories and ordering cells based on gene expression patterns [46].

## 3. Results

### 3.1. Rapid Immune Responses across Different Organs after SARS-CoV-2 Infection

The spike of glycoprotein on the SARS-CoV-2 virus envelope exhibits a specific affinity for the ACE2 receptor, a functional distinctive membrane receptor on host cells [47]. According to the data sourced from the Human Protein Atlas (https://www.proteinatlas.org/ENSG00000130234-ACE2/summary/rna, accessed on 4 January 2024), ACE2 demonstrates significant expression in extrapulmonary organs, such as the liver, heart, brain, and kidneys, which indicates a high risk of SARS-CoV-2 infection (Table S1). In this study, to explore more about the inter-relationship of SARS-CoV-2 infection across different organs, four bulk RNA-seq datasets from lung, choroid plexus organoids, adult cardiomyocytes, and human embryonic kidney cells were firstly used to investigate the transcriptional heterogeneity and homogeneity [25,26,27,28].

**Figure 1:**
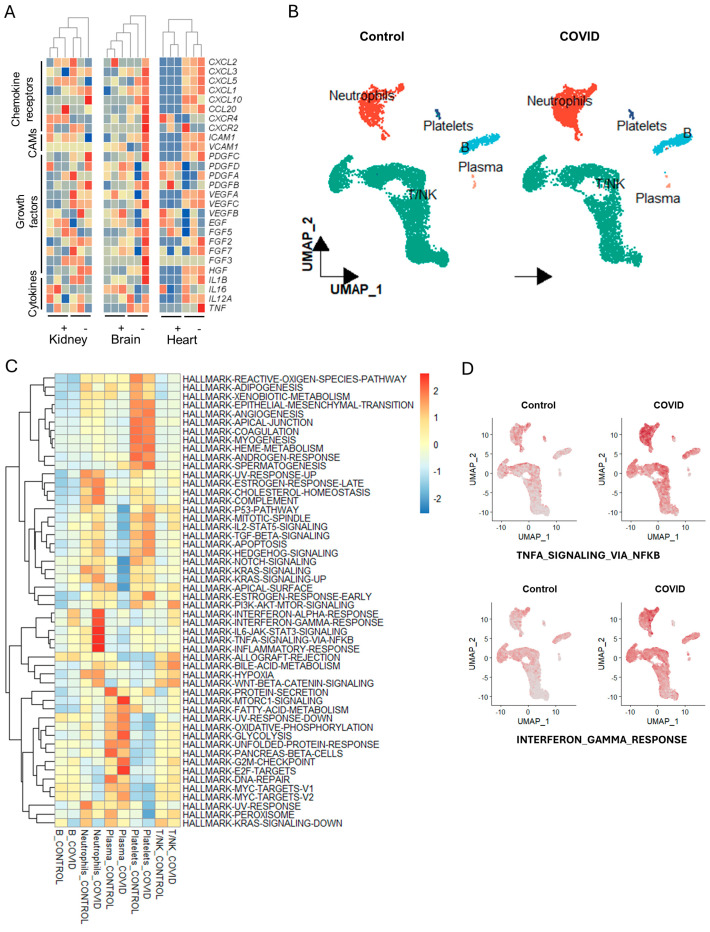
(**A**) Heatmap of immune response-related genes in adult cardiomyocytes, choroid plexus organoids, and human embryonic kidney cells. Blue indicates low expression, while orange indicates high expression. +/−, with/without SARS-CoV-2 infection. (**B**) UMAP plot of cell cluster in COVID and control group. (**C**) Pathway enrichment analysis with HALLMARK database in different cell types between COVID and control group. (**D**) Feature plot of key immune pathways in different groups.

Currently, with no perfect drugs for COVID-19 treatment, vaccination works as the most effective way to prevent viral infection [48,49]. Multiple genes and pathways work coordinately for immune activities. Herein, we used the GSEA to explore the enrichment of immune-related pathways in the early stage of SARS-CoV-2 infection. For the lung tissues, primary immunodeficiency and viral myocarditis were highly upregulated after COVID infection, which indicates that SARS-CoV-2 could cause systematic immune effect and cardiac injury (Table S2). These are consistent with previous clinical studies, in which COVID-19 patients with a compromised immune system had a higher risk of cardiac injury [50,51]. For the choroid plexus, cytokine–cytokine receptor interaction, chemokine signaling pathway, and leukocyte trans-endothelial migration were significantly upregulated after SARS-CoV-2 infection (Table S2), which have been reported as biomarkers for prognosis in COVID-19 patients [52,53]. Adult cardiomyocytes also exhibited enhanced immune and inflammation responses, characterized by a highly enriched T cell receptor signaling pathway, Toll-like receptor signaling pathway, and chemokine signaling pathway (Table S2). Similarly, human embryonic kidney cells also exhibited cytokine–cytokine receptor interaction after SARS-CoV-2 infection (Table S2). We conducted a supervised comparison of gene expressions for chemokines, chemokine receptors, cell adhesion molecules (CAMs), growth factors, and cytokines. The result indicates that these markers exhibited higher expression across these organs following SARS-CoV-2 infection (Figure 1A), indicating a rapid systemic tissue-resident immune response. To further investigate the infiltrating immune response, we analyzed the single-cell RNA-seq (scRNA-seq) data from whole blood samples obtained from both healthy individuals and COVID-19 patients (Figure 1B) [29]. A pathway enrichment analysis showed that there was a significant increase in immune response within the neutrophils, such as TNFα, IFNγ, and IL-6, indicating the crucial role of neutrophils in immune defense mechanisms (Figure 1C,D) [54]. Collectively, these findings indicate that SARS-CoV-2 infection can lead to rapid systemic immune responses across various organs. 

### 3.2. SARS-CoV-2 Infection Induces Organ-Specific Transcriptional Profiling

**Figure 2:**
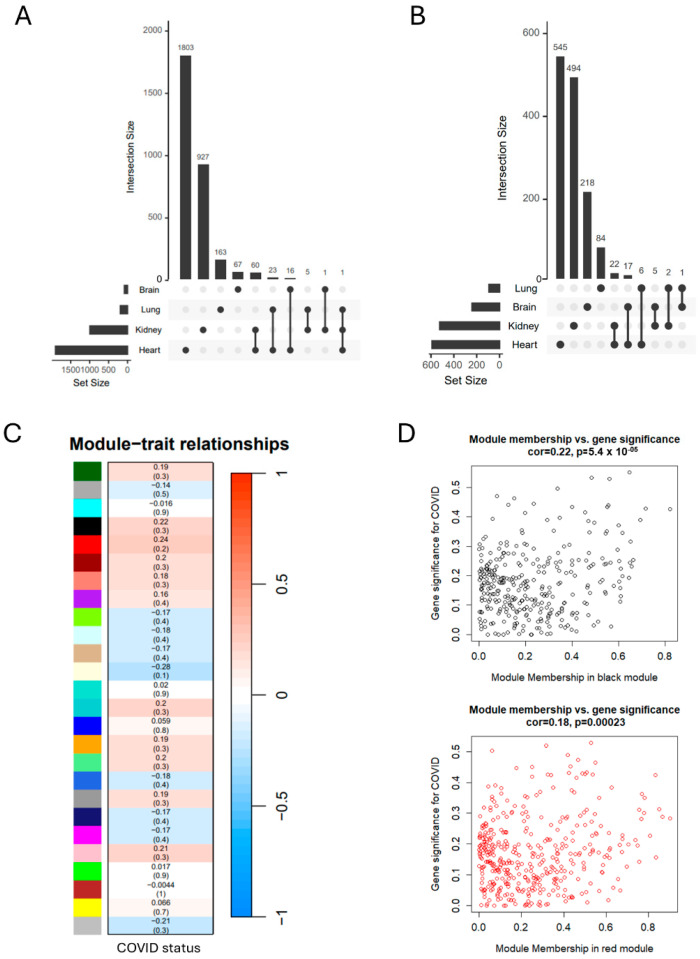
WGCNA analysis across different organs. (**A**) Upset plot of common upregulated DEGs in different organs. (**B**) Upset plot of common downregulated DEGs in different organs. (**C**) Relationship of module genes with COVID status. (**D**) Correlation of key module genes with COVID status.

To explore the common gene activation across diverse organs during COVID infection, we conducted a comparative analysis between the SARS-CoV-2 infection and control groups in each organ. Subsequently, we filtered the significantly different expressed genes (DEGs) with a cut-off criterion of |Fold change| > 1.5 and *p*-value < 0.01. The number of differentially expressed genes varied across the organs, and we did not observe commonly up- or downregulated DEGs based on the upset plot (Figure 2A,B). Next, we combined the raw count data across these organs and conducted a weighted gene co-expression network analysis (WGCNA). The results revealed that the core module genes had a low correlation with COVID-19 status (Figure 2C,D), suggesting the existence of potentially tissue-specific expression patterns.

**Figure 3:**
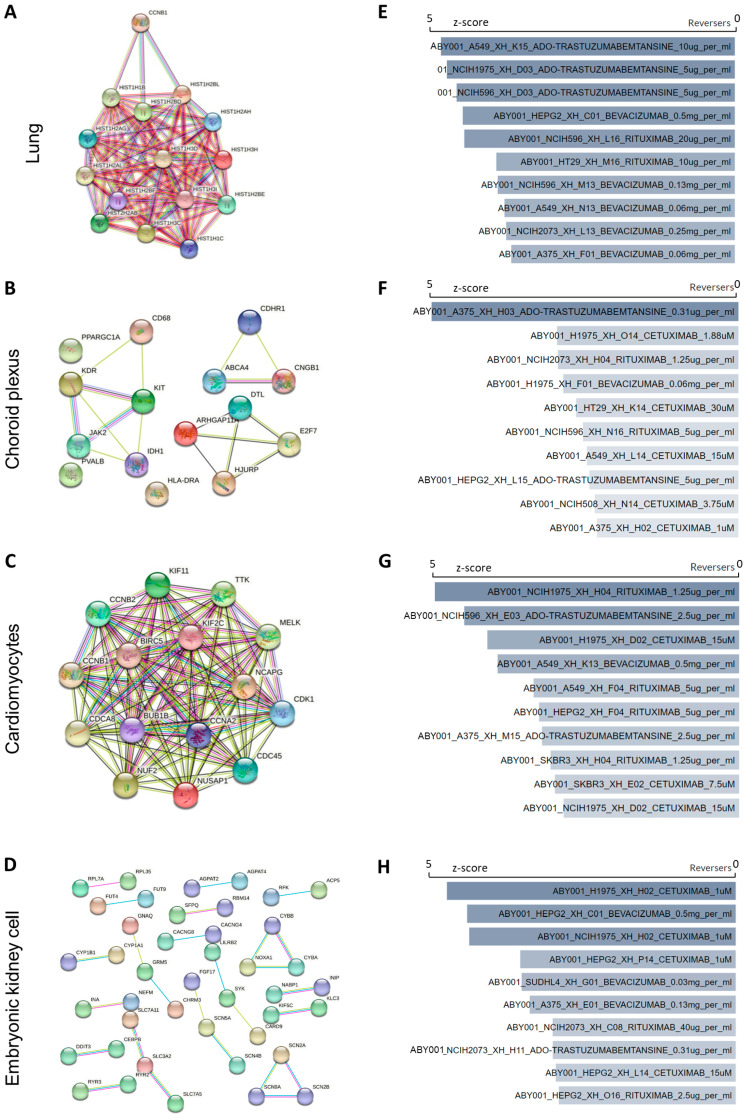
PPI network and predicted candidate antibodies. (**A**–**D**) PPI network analysis of top 15 hub genes by STRING. (**E**–**H**) Prediction of candidate antibody by LINCS l1000 antibody perturbations database. Ranking by z-score.

To further investigate the tissue-specific patterns, we imported the DEGs from each dataset into STRING to construct a protein–protein interaction network. Cytohubba was incorporated with PPI to identify the hub genes for each dataset. The central elements were ranked with the maximal clique centrality (MCC) method, and the top 15 genes were regarded as hub genes (Table S3). For the lung tissue, most of the PPI genes were from the HIST1H family, indicating an association with elevated chromatin assembly processes and a systemic immune response (Figure 3A and Table S3) [55]. The activation of JAK2 within the choroid plexus implied the initiation of the interleukin-35-mediated signaling pathway (Figure 3B and Table S3) [56]. Furthermore, the perturbation of CNGB1, which is involved in olfactory nerve maturation, suggested a potential structure impairment following SARS-CoV-2 infection [57]. For the cardiomyocytes, the PPI network mainly focused on ATP production (CCNB1 and CDK1), indicating a high energy demand (Figure 3C and Table S3). For the human embryonic kidney cells, multiple genes, including CYP1A1, CYBB, NABP1, and KIF5C, were involved (Figure 3D and Table S3), indicating a broad immune response [58,59]. Similarly, there was no overlap of hub genes across different organs. These results indicate that SARS-CoV-2 infection could lead to heterogeneous transcriptional profiling.

### 3.3. Characterization of Hub Genes–Disease Association and Prediction of Candidate Drugs and Antibodies

Transcriptional dysregulation always leads to various pathological phenotypes. Herein, we used DisGeNET to predict the potential associations between hub genes and other complications. Interestingly, apart from respiratory defects, we observed a consistent enrichment of brain cancers related diseases such as glioblastoma and ganglioglioma across these organs (Table S4). Clinical studies have reported that 80% of COVID-19 hospitalized patients had neurological symptoms and predominant manifestations including acute encephalopathy, coma, and stroke. It is demonstrated that SARS-CoV-2 could potentially damage the brain in multiple ways. The virus is capable of attacking specific brain cells, reducing the blood supply to brain tissue, and inducing the production of immune molecules that damage brain cells [60]. However, further studies should be performed to investigate the consequences of COVID-related brain damage. Furthermore, diseases related to the digestive system have also been documented in these organs, such as esophagus diseases, hyperinsulinism, and neoplasms of the stomach and colon. It is reported that more than half of COVID-19 patients are at a higher risk of developing hyperglycemia, and approximately 33% of patients developed diabetic ketoacidosis [61,62]. However, there is limited study on the impact of COVID-19 on the other digestive organs. Prolonged clinical observation is crucial in refining medical strategies, enhancing patient care, and ultimately mitigating the impact of the pandemic.

Currently, effective drug treatment for COVID-19 treatment is still limited. Clinical trials have indicated that remdesivir is effective in reducing the recovery time and mitigating respiratory tract infection for hospitalized COVID-19 adults. The mechanism of remdesivir is suppressing the viral RNA-dependent RNA polymerase (RdRp) [63]. Herein, we predicted potential drugs based on the hub genes from different datasets (Table S5). Mechanistically, these drugs can either attenuate inflammation or impede viral entry, representing strategic approaches in new drug design. We found that some of the drugs showed promising effects in in vivo experiments and are under clinical trials, such as etoposide, niclosamide, valsartan, and dasatinib [64,65,66,67]. Moreover, we identified some potential drugs that could be beneficial for COVID-19 recovery. Mefloquine, a drug used for malaria, worked well as an anti-SARS-CoV-2 entry inhibitor in vitro [68]. LY-294002, an inhibitor of PI3K, could effectively decrease the replication and DNA synthesis of Marek’s disease virus, which is also regarded as a potential modulator for MERS-CoV infection [69]. Terfenadine, a histamine H1 receptor antagonist, could induce micropinocytosis [70]. Researchers have suggested investigating its role in inhibiting SARS-CoV-2 endocytosis [71]. Piroxicam, an FDA-approved nonsteroidal anti-inflammatory drug, showed promising antiviral activity against NRC-03-nhCoV in vitro, which is suggested to be used in combination with azithromycin for COVID-19 patients [72]. Interestingly, testosterone was also regarded as a candidate agent. Recent clinical studies revealed that male COVID-19 patients with lower testosterone levels were more likely to experience severe symptoms [73,74]. Additionally, we utilized the LINCS L1000 Antibody Perturbations Database to predict candidate antibodies for each organ. We selected the top 10 reversers that could potentially reverse the transcriptional dysregulation caused by SARS-CoV-2 infection (Figure 3E–H). Notably, bevacizumab was significantly enriched in all the organs analyzed, indicating its potential to mitigate the systemic effects of COVID-19. Previous clinical studies have shown that bevacizumab improves oxygenation and reduces the duration of oxygen support, demonstrating its clinical efficacy [75]. However, more in vitro and in vivo studies are necessary to further validate its effects.

### 3.4. Kidney Dysfunction Results in Compromised Immune Response

**Figure 4:**
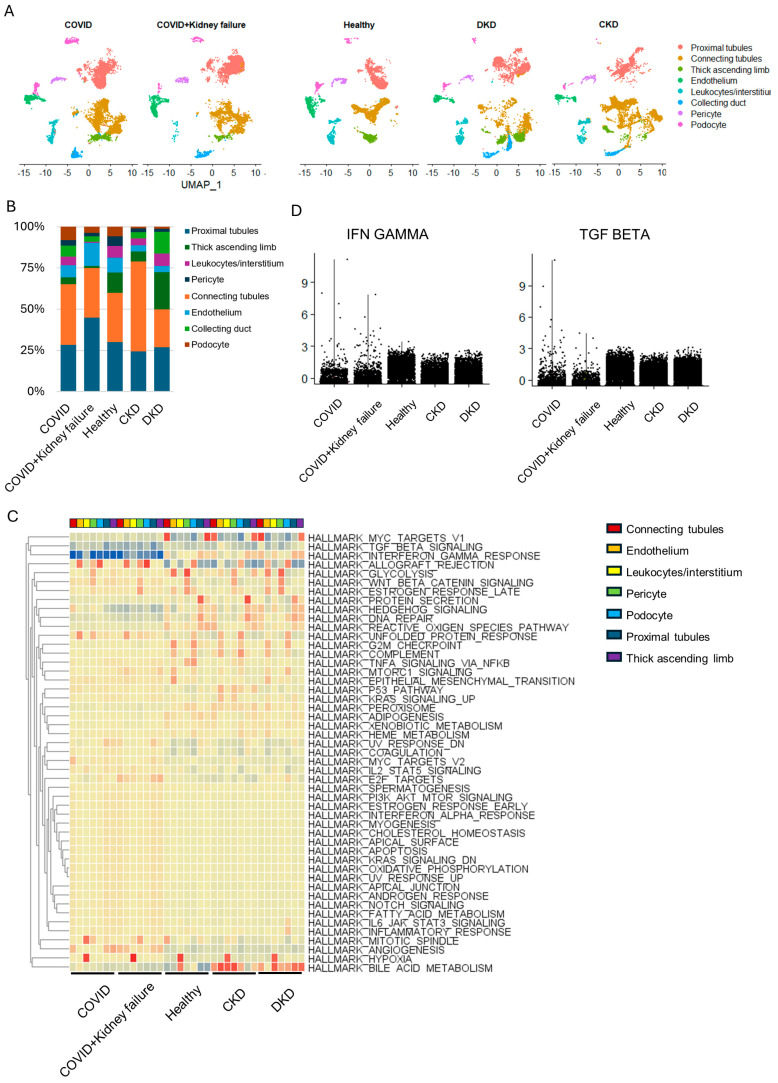
Single-cell RNA-seq analysis of kidney samples from different patients. (**A**) UMAP plot of specific cell-type clusters. (**B**) Cell proportion in different groups. (**C**) Gene set enrichment analysis of metabolic features across different cell types of different groups. (**D**) Violin plot of IFNγ and TNFα pathway scores in different groups.

COVID-19 can lead to respiratory failure and other organ failure due to a hyperinflammatory response [76]. The relationship between kidney failure and COVID-19 is complex and involves a combination of direct viral effects, systemic inflammation, hypoxia, coagulation abnormalities, and treatment-related factors [19]. It underscores the importance of monitoring kidney function in COVID-19 patients and implementing strategies to prevent and manage kidney complications. Chronic kidney disease (CKD) and diabetic kidney disease (DKD) are two prevalent and interconnected conditions that significantly impact overall health, leading to kidney failure. The interplay between CKD, DKD, and immune activity is complex and multifaceted. Both conditions contribute to an altered immune response, rendering patients more susceptible to infections, impaired wound healing, and chronic inflammation [77,78]. To elucidate the complex effects of kidney dysfunction on immune activity, we integrated single-cell RNA-seq datasets from three COVID-19 patients, three COVID-19 patients with concurrent kidney failure, three CKD patients, three DKD patients, and six healthy controls for further analysis (Figure 4A) [30,31]. Although there was a slight change in the ratio of different epithelial cells, the overall proportion of epithelial cells was not significantly affected (Figure 4B). There was a decrease in leukocytes in patients with kidney failure, CKD, or DKD. Unlike the rapid immune response during the early infection stage, a gene set enrichment analysis showed that patients with long-term COVID-19 exhibited significantly reduced immune activity (Figure 4C), especially in IFNγ and TGFβ (Figure 4D), while other metabolic pathways were not significantly changed. Additionally, we observed reduced immune activity in COVID-19 patients with kidney failure compared to those without kidney failure. Moreover, patients with CKD and DKD exhibited a compromised immune response compared to healthy individuals (Figure 4D). These results indicate that long-term kidney dysfunction could render patients more susceptible to COVID-19 infection. 

### 3.5. Evaluate the Correlation of Kidney Failure on Lung Function in COVID-19 Patients

**Figure 5:**
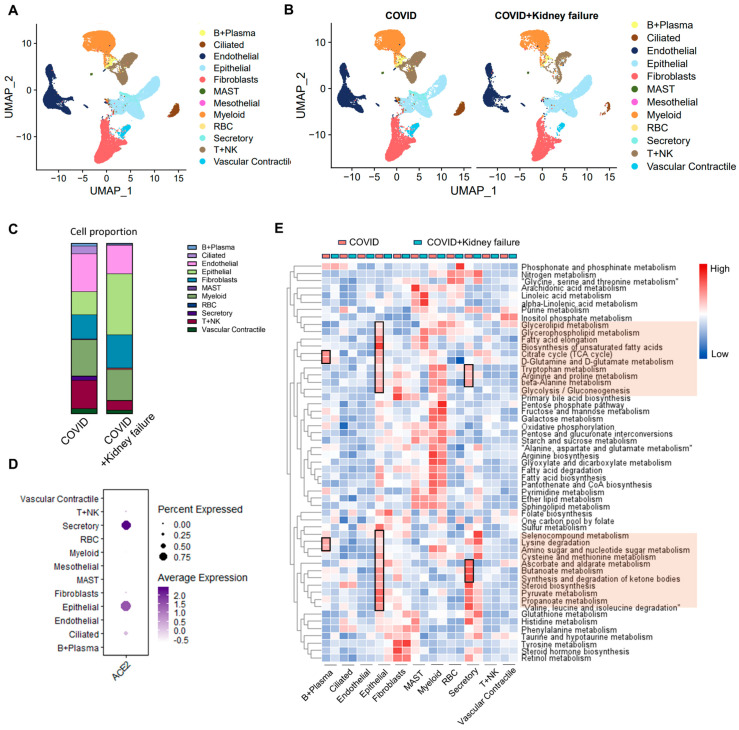
Single-nucleus RNA-seq analysis of lung samples in COVID-19 patients. (**A**) UMAP plot of specific cell-type clusters. (**B**) UMAP plot of cell types in COVID and COVID + kidney failure groups. (**C**) Cell proportion in different groups. (**D**) Dot plot of ACE2 expression in different cell types. (**E**) Gene set enrichment analysis of metabolic features in different cell types between COVID and COVID + kidney failure groups. Red color indicates upregulation in COVID group. Black rectangles and red boxes indicate the highly expressed pathways in the COVID group.

**Figure 6:**
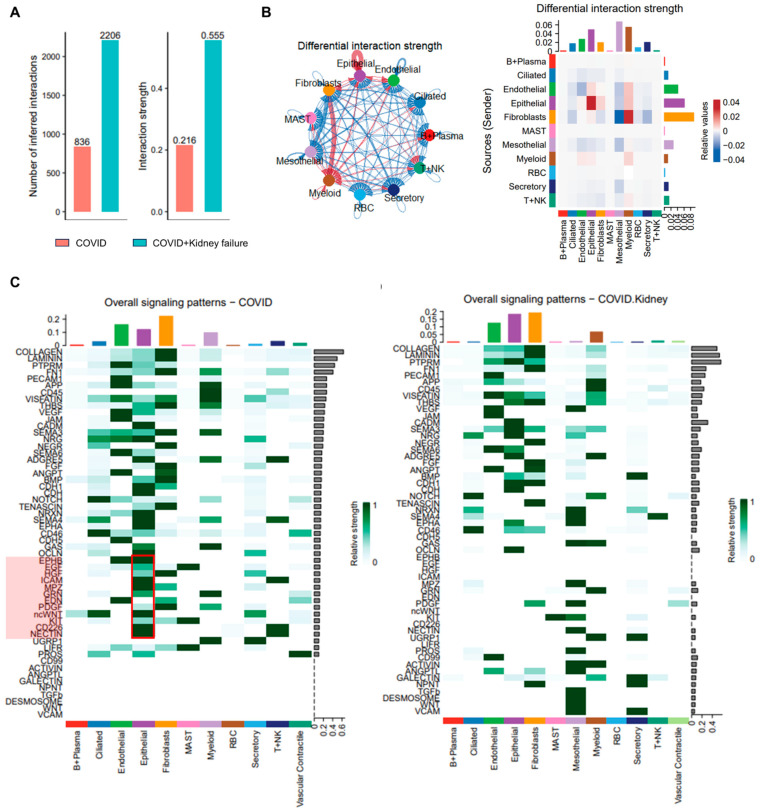
Cell–cell interactions of lung samples in COVID-19 patients. (**A**) Overview of the number and strength of cell–cell interactions in different groups. (**B**) Chord plot (**left**) and heatmap of cell–cell interaction in different groups. Red color indicates upregulation in COVID group. Blue color indicates upregulation in COVID + kidney failure group. (**C**) Comparison of overall signal pattern in different groups. Red color indicates upregulation in COVID group. Red boxes indicate the highly expressed signaling patterns in the COVID group.

To elucidate the systemic correlation of kidney dysfunction and COVID-19, we re-analyzed the single-nucleus RNA-seq data obtained from lung, liver, and heart samples from three COVID-19 patients and another three COVID-19 patients with concurrent kidney failure (Table S5) [31]. After eliminating doublets and low-quality cells from the lung samples, a total of 12 cell types were identified in the lung samples, including epithelial cell, endothelial cells, myeloid cells, and B, T, and NK cells (Figure 5A,B). The cellular composition showed that the COVID group exhibited fewer epithelial cells and a greater abundance of endothelial cells, as well as T + NK cells (Figure 5C). Additionally, ACE2 was mainly expressed in secretory, epithelial, and ciliated cells, suggesting that these cell types are more susceptible to infection (Figure 5D). VISION was employed to calculate the metabolic activities in various cells within the two groups [42,79]. We observed highly increased metabolic features in the epithelial cells of the COVID group, with upregulation of pathways related to amino acids, fatty acids, and glucose, indicating a more activated immune response (Figure 5E). This was further supported by the relatively higher metabolic activity observed in B + plasma cells and secretory epithelial cells in the COVID group. However, kidney failure could affect these metabolic features, implying mild immune activity. Moreover, other metabolic activities were not significantly altered in other cell types. We further analyzed the global cell–cell communication using CellChat. The COVID + kidney failure group showed higher inferred interactions with greater overall strength compared to the COVID group, suggesting more complex cellular activity in COVID-19 patients with kidney failure (Figure 6A). Moreover, the epithelial interactions were less active in the COVID + kidney failure group (Figure 6B), especially inflammation-related signals, such as HGF, ICAM, WNT, CD226, and NECTIN (Figure 6C), indicating a weakened immune response. These results suggest that kidney failure could impact the ability of lung epithelial cells to mount an effective immune response to COVID-19 infection, potentially leading to a less effective defense against the virus.

### 3.6. COVID-19 Patients with Kidney Failure Exhibited De-Differentiation of AT1 Cells

**Figure 7:**
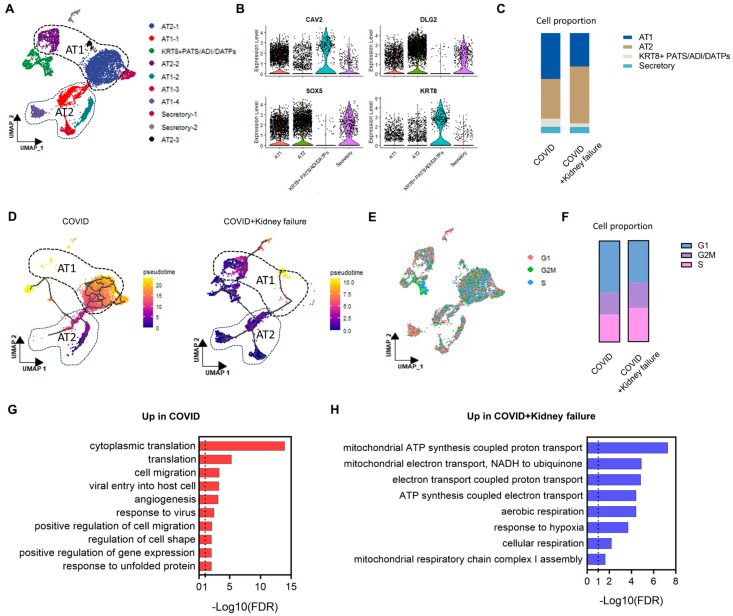
Subcluster analysis of epithelial cells of lung samples. (**A**) UMAP plot of specific cell-type clusters. (**B**) Violin plot of specific cell-type marker expressions. (**C**) Cell proportion of sub-epithelial cells in different groups. (**D**) Trajectory analysis of cellular transition in different groups. (**E**) Individual cells were scored for their cell cycle genes (G1, G2M, and S). (**F**) Cell proportion by proliferation stage in different groups. (**G**) Enrichment of DEGs upregulated in COVID group. (**H**) Enrichment of DEGs upregulated in COVID + kidney failure group.

As ACE2 was mainly expressed in secretory and epithelial cells (Figure 5D), we isolated these epithelial cells for further analysis. The epithelial cells could be divided into alveolar type 1 cells (AT1), alveolar type 2 cells (AT2), and KRT8 + PATS (KRT8+ pre-alveolar type 1 transitional cell state) cells based on specific marker expressions (Figure 7A,B). In the COVID + kidney failure group, there was a relatively higher proportion of AT2 cells and a lower proportion of AT1 cells (Figure 7C). Furthermore, a trajectory analysis revealed that the AT1 cells in the COVID + kidney failure group were less mature (Figure 7D). These results suggest de-differentiation of AT1 cells in COVID-19 patients with kidney failure, potentially resulting in an impaired gas exchange ability. A cell cycle analysis showed that the COVID + kidney failure group contained more S-stage cells but less G1-stage cells (Figure 7E,F), indicating a higher proliferation activity and less matured epithelial cell state. We further filtered the DEGs between the COVID and COVID + kidney failure groups with |log2FC| > 0.25 and *p*-value < 0.01. The functional annotation of up/down DEGs were analyzed on DAVID. The cytoplasmic translation, viral entry into the host cell, response to the virus, and regulation of cell migration pathways were highly enriched in the COVID group (Figure 7G), implicating a more active immune response. However, the COVID + Kidney failure group exhibited a cellular stress state with upregulation of the mitochondrial pathways (Figure 7H). A previous clinical study reported that COVID-19 patients undergoing hemodialysis exhibited a more significant reduction in serum inflammatory cytokines compared to other COVID-19 patients [65]. This finding is consistent with our results, and one possible reason could be the changes in cell proportions and lung epithelial de-differentiation.

### 3.7. COVID-19 Patients with Kidney Failure Exhibited Increased Fibrosis in Multiple Organs

**Figure 8:**
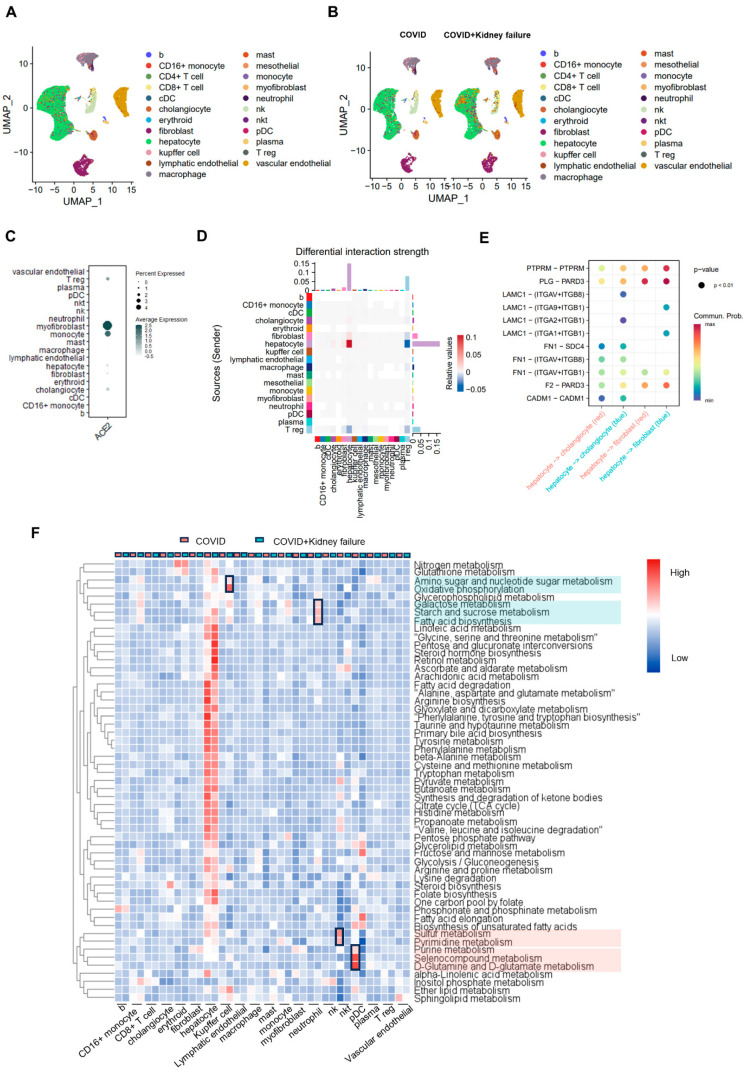
Single-nucleus RNA-seq analysis of liver samples in COVID-19 patients. (**A**) UMAP plot of specific cell-type clusters. (**B**) UMAP plot of cell types in COVID and COVID + kidney failure groups. (**C**) Dot plot of ACE2 expression in different cell types. (**D**) Chord plot of cell–cell interaction in different groups. Red color indicates upregulation in COVID group. Blue color indicates upregulation in COVID + kidney failure group. (**E**) Ligand-receptor comparison in different groups. Red color indicates upregulation in COVID group. Blue color indicates upregulation in COVID + kidney failure group. (**F**) Gene set enrichment analysis of metabolic features in different cell types. Red color indicates upregulation in COVID group. Blue color indicates upregulation in COVID + kidney failure group. Black rectangles indicate the significant changes pathways. Red boxes indicate the highly expressed pathways in the COVID group. Turquoise boxes indicate the highly expressed pathways in the COVID + kidney failure group.In the heart, ACE2 was mainly expressed in the pericyte, with slight expression observed in cardiomyocytes (Figure 9A–C). COVID-19 patients with kidney failure results in increased interactions of LAMC1/LAMA4/LAMA2-integrin and COL4A1/COL4A2/COL4A5-intergin, especially between cardiomyocyte and fibroblast and pericytes and endothelial cells, indicating a potential endothelial dysfunction and thrombosis (Figure 9D,E) [80,81]. This was further supported by upregulated fibrosis markers of VIM, FN1, and PDGFRB in the fibroblast and endothelial cells within the COVID + kidney failure group (Figure 9F) [82,83]. Furthermore, we noted relatively lower metabolic activity in the neutrophils and NKT cells of the COVID + kidney failure group, including fatty acid and glutamine metabolism (Figure 9G).

We next analyzed the effects of kidney failure on other organs (liver and heart) of COVID-19 patients. Liver and heart samples were obtained from the same COVID-19 patients. The cell types for heart and liver were based on automate and manual annotation of marker genes [31]. COVID-19 patients with kidney failure did not exhibit altered composition of liver cells (Figure 8A,B). Moreover, ACE2 expression was predominantly observed in myofibroblast cells, with lower expression observed in hepatocytes and cholangiocytes (Figure 8C). In the COVID + kidney failure group, hepatocytes exhibited higher levels of cell–cell communication compared to other cell types (Figure 8D), with more active LAMC1–integrin and FN1–integrin interactions in hepatocyte-to-cholangiocyte and hepatocyte-to-fibroblast interactions (Figure 8E), indicating extracellular matrix modeling and cell migration. However, the metabolic features were not significantly affected in COVID-19 patients with kidney failure (Figure 8F). Although there was a slight change observed in the metabolic features of neutrophils, NKT cells, and pDC cells, it was not significant enough to impact liver function (Figure 8F).

**Figure 9:**
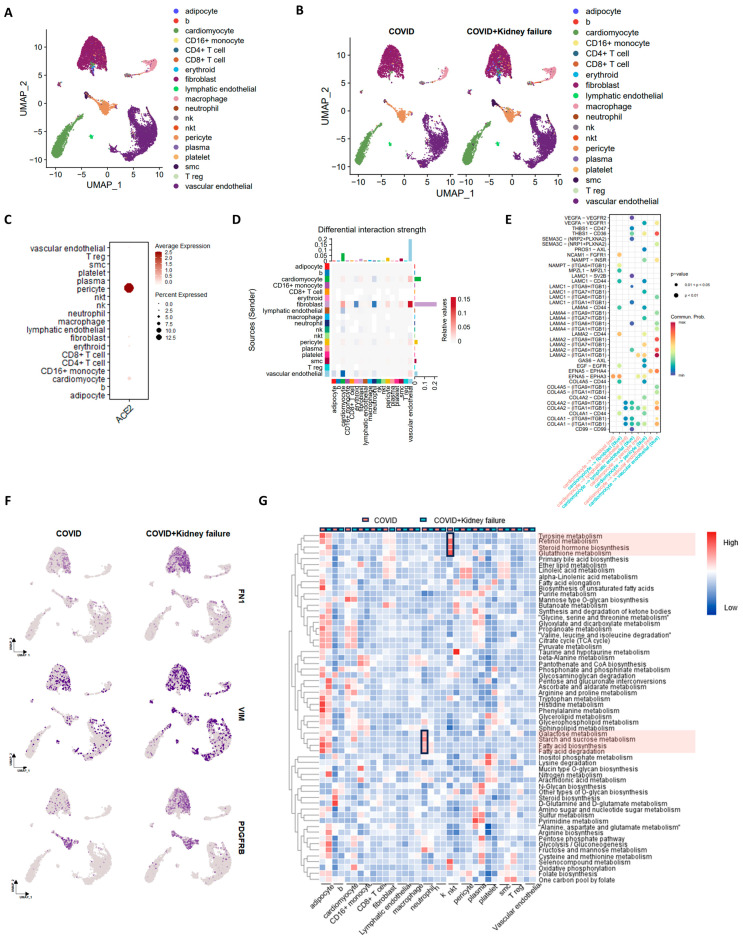
Single-nucleus RNA-seq analysis of heart samples in COVID-19 patients. (**A**) UMAP plot of specific cell-type clusters. (**B**) UMAP plot of cell types in COVID and COVID + kidney failure groups. (**C**) Dot plot of ACE2 expression in different cell types. (**D**) Chord plot of cell–cell interaction in different groups. Red color indicates upregulation in COVID group. Blue color indicates upregulation in COVID + kidney failure group. (**E**) Ligand-receptor comparison in different groups. Red color indicates upregulation in COVID group. Blue color indicates upregulation in COVID + kidney failure group. (**F**) Feature plots of fibrosis markers VIM, FN1, and PDGFRB in different groups. (**G**) Gene set enrichment analysis of metabolic features in different cell types. Red color indicates upregulation in COVID group. Black rectangles and red boxes indicate the highly expressed pathways in the COVID group.

**Figure 10:**
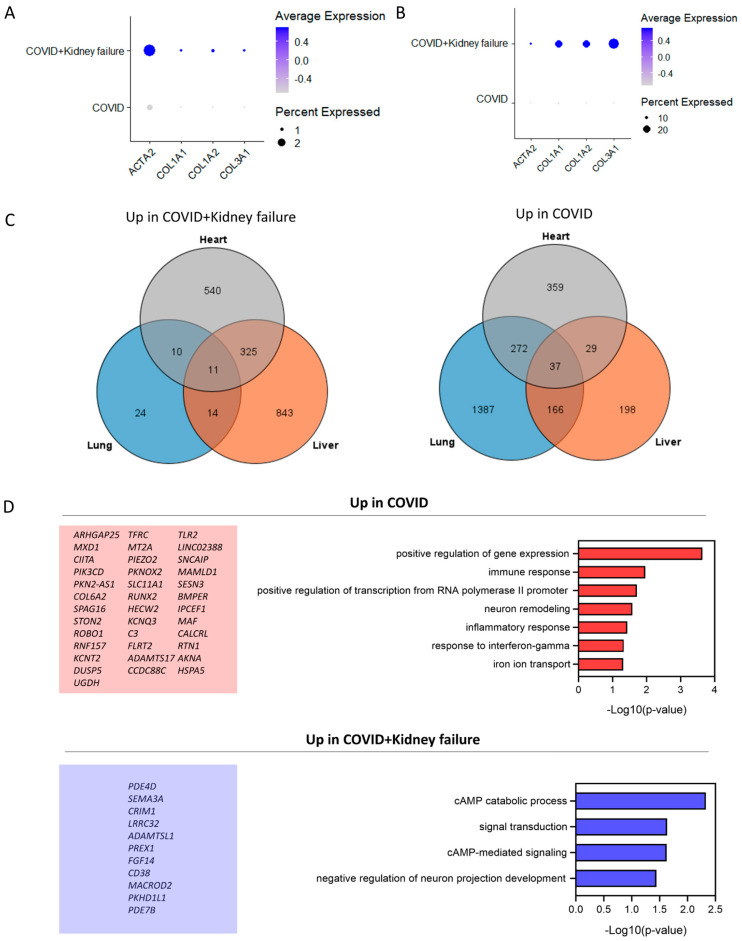
Comparison of up/down DEGs in different organs. (**A**) Dotplot of fibrosis genes in hepatocytes. (**B**) Dotplot of fibrosis genes in lung epithelial cells. (**C**) Identification of common up-/downregulated DEGs in different datasets. (**D**) Enrichment of common up-/downregulated DEGs in COVID group.

Next, to further confirm the fibrosis in hepatocytes (Figure 10A) and lung epithelial cells (Figure 10B), we compared the key fibrosis genes with a dotplot. Surprisingly, these genes were highly increased in these two cell types of COVID-19 patients with kidney failure, indicating broad fibrosis. We did not observe significant compromise of immunity in the hepatocytes and cardiomyocytes (Figure S1). Additionally, we filtered the up/down DEGs in the lung, liver, and heart between the COVID and COIVD + kidney failure groups (Figure 10C). In the COVID + kidney failure group, there were 11 commonly upregulated and 37 commonly downregulated genes observed across these organs (Figure 10D). A gene enrichment analysis revealed that the COVID + kidney failure group exhibited upregulation of cAMP signaling, possibly because of disrupted homeostasis. However, a decreased immune response was observed in all these organs. 

## 4. Discussion

COVID-19 can manifest with a range of symptoms that extend beyond the respiratory system. Clinical investigations have proved the extensive effect of COVID-19 on cardiovascular disease, diabetes, acute kidney injury, and even brain structure [8,9,84,85]. However, most of the research is based on clinical observations, and there is insufficient understanding of the molecular mechanisms underlying the impact of COVID-19 on other organs. 

In terms of homogeneity, analyses of both bulk RNA-seq data and single-cell RNA-seq data have unveiled a consistent upregulation of the immune response in multiple organs and blood after SARS-CoV-2 infection, suggesting systemic immune activity. We performed a comparative analysis between SARS-CoV-2 infection and control groups in lung, choroid plexus organoids, adult cardiomyocytes, and kidney embryonic cells. The DEGs varied extensively across organs, with no common distinct DEGs, indicating an organ-specific transcriptional profile. We further developed a PPI network and identified the hub genes. Interestingly, the hub gene–disease association analysis showed that there was a conservation enrichment of brain cancer-related diseases across these organs. A previous study by magnetic resonance imaging and cognitive analysis revealed deleterious neurological effects in COVID-19 patients [85]. However, it is still unclear whether these abnormalities are caused by neuroinflammation or direct viral infection on brain cells.

Kidneys perform their essential roles in maintaining overall health and homeostasis in the body. Research has revealed a close relationship between declining kidney function and the risk of mortality from COVID-19. Individuals with kidney failure face particularly high mortality rates [19]. Studies have indicated that SARS-CoV-2 infection can trigger the release of an inflammatory cytokine storm, which could be the primary cause of a worsened condition and even death in patients [86]. It has been reported that hemodialysis patients with COVID-19 often present with mild clinical symptoms, possibly due to compromised cellular immune function and the inability to mount a cytokine storm. This results in a longer time required to clear the virus and a persistent virus shedding duration [87]. In our study, we observed similar results with decreased metabolic activity in lung epithelial cells. Additionally, COVID-19 patients with kidney failure exhibited a dedifferentiation of AT1 cells, potentially impairing the epithelial immune response and gas exchange ability. Moreover, there was a slight decrease in immune response observed in the liver and heart. Less cytokine storm is beneficial for patient survival but also indicates more time to recover. Considering the potential interaction between kidney failure and the immune response, more understanding is needed regarding how vaccination, including additional and booster doses, reduces the risk of severe illness in patients with kidney disease. Clinical trials are necessary to identify strategies to minimize the risk of adverse outcomes, such as the development of chronic kidney disease (CKD), kidney failure, or thrombosis.

The main limitation of this study is the lack of in vivo or in vitro validation. To enhance the robustness of the RNA-seq result, it is essential to integrate experimental approaches and clinical observation to validate the findings. However, this is a common challenge for COVID-19 studies. The effects of SARS-CoV-2 infection have been reported in lung organoid, intestine organoid, and kidney organoid [88,89,90]. Organoid models will serve as valuable platforms to study the COVID effects on other organs, as well as drug screening and vaccine development. Another constraint lies in the limited size of our analyzed sample. This investigation serves as an initial exploration, offering a preliminary insight into potential organ- and cell-type-specific metabolic alterations post-COVID infection. Future studies with larger sample cohorts will be essential to validate and extend our findings.

## Data Availability

All the data are deposited in the GEO and can be search based on the GEO number mentioned in the Section 2. We have made all relevant scripts and data used to produce the results available in a public repository. The scripts can be accessed at GitHub (https://github.com/lipai0303/COVID-intergrated-analysis, accessed on 30 July 2024).

## Supplementary Materials

The following supporting information can be downloaded at https://www.mdpi.com/article/10.3390/life14080960/s1, Figure S1. Pathway enrichment analysis with HALLMARK database in hepatocyte and cardiomyocyte types between COVID and COVID + Kidney failure group; Table S1. Expression of ACE2 in different organs; Table S2. GSEA analysis of SARS-CoV-2 infection in different organs; Table S3. Top 15 hub genes by PPI network analysis in each dataset; Table S4. Top 10 gene associated diseases by DisGeNET; Table S5. Prediction of candidate drugs by DSigDB; Table S6. Metadata of scRNA-seq datasets used in this study.
