# Supplementary material for: Integrated Transcriptomic Analysis Reveals Reciprocal Interactions between SARS-CoV-2 Infection and Multi-Organ Dysfunction, Especially the Correlation of Renal Failure and COVID-19
Source: Life (Basel). 2024 Jul 30;14(8):960. doi: 10.3390/life14080960 (PMC11355357; doi:10.3390/life14080960)
Supplement: Supplementary file 1 [file life-14-00960-s001.zip › Figure S1, Table S1, S3-S5.pdf]

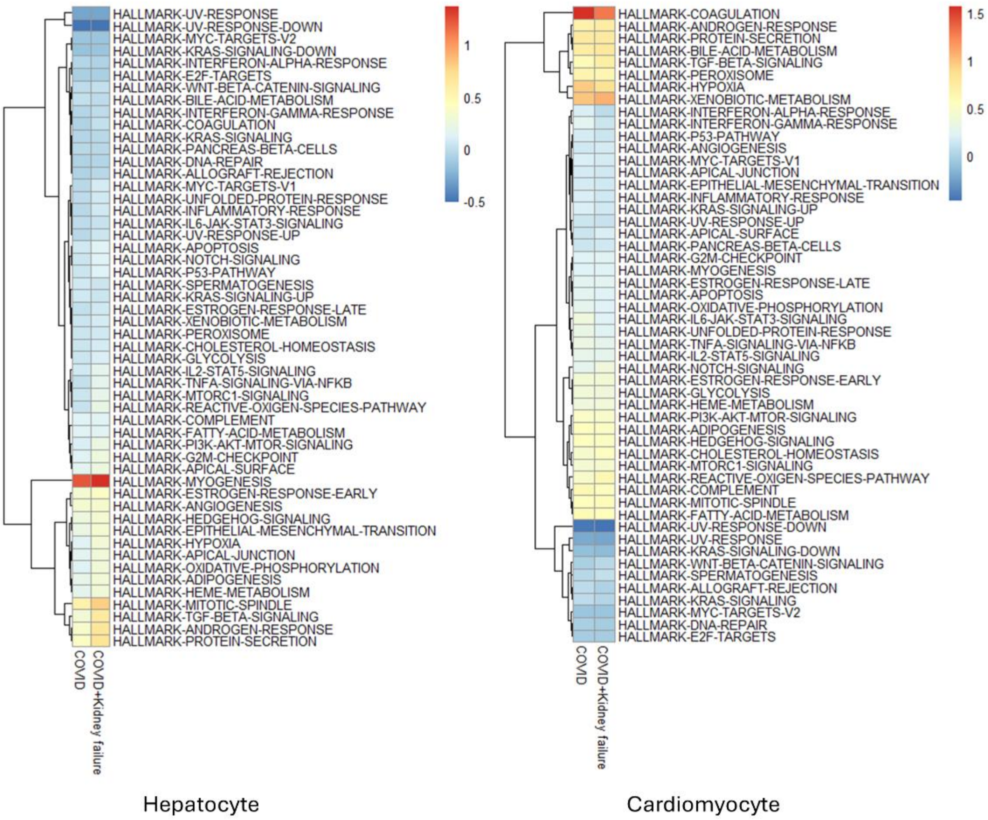

Figure S1. Pathway enrichment analysis with HALLMARK database in hepatocyte and cardiomyocyte types between COVID and COVID+Kidney failure group.

Table S1. Expression of ACE2 in different organs.

Organ	nTPM	Sample number
Lung	0.8	9
Liver	5.2	10
Choroid plexus	5.5	2
Heart muscle	29.5	4
Salivary gland	0.7	3
Colon	9.8	13
Pancreas	1.9	2
Kidney	11.5	57
Adrenal gland	0.5	3

Table S3. Top 15 hub genes by PPI network analysis in each dataset.

Rank	Gene	Score	Up/Down
**GSE150316**			
1	HIST2H2AB	1.78E+08	Up
2	HIST1H3D	1.78E+08	Up
3	HIST1H2AH	1.78E+08	Up
4	HIST1H3I	1.78E+08	Up
4	HIST1H3H	1.78E+08	Up
6	HIST1H3C	1.78E+08	Up
7	HIST1H1C	1.67E+08	Up
8	HIST1H2BE	1.67E+08	Up
8	HIST1H2AG	1.67E+08	Up
8	HIST1H2AL	1.67E+08	Up
11	HIST1H2BL	9.44E+07	Up
12	HIST1H2BD	9.44E+07	Up
13	HIST1H1B	8.35E+07	Up
14	HIST1H2BF	8.35E+07	Up
15	CCNB1	4.75E+07	Up
**GSE157852**			
1	KIT	15	Down
2	KDR	12	Down
3	JAK2	9	Down
3	IDH1	9	Down
5	ARHGAP11A	7	Down
5	DTL	7	Up
5	HJURP	7	Up
5	PVALB	7	Down
5	CDHR1	7	Down
5	CD68	7	Up
11	HLA-DRA	6	Down
11	PPARGC1A	6	Down
11	E2F7	6	Up
11	ABCA4	6	Down
11	CNGB1	6	Down
**GSE151879**			
1	BUB1B	3.61E+98	Up
1	CCNA2	3.61E+98	Up
1	CDK1	3.61E+98	Up
1	NCAPG	3.61E+98	Up
1	KIF11	3.61E+98	Up
1	CDC45	3.61E+98	Up
1	CCNB1	3.61E+98	Up
1	BIRC5	3.61E+98	Up

1	NUF2	3.61E+98	Up
1	TTK	3.61E+98	Up
1	NUSAP1	3.61E+98	Up
1	CCNB2	3.61E+98	Up
1	CDCA8	3.61E+98	Up
1	MELK	3.61E+98	Up
1	KIF2C	3.61E+98	Up
**GSE189706**			
1	CYBB	3.4E+08	Up
1	SCN2A	3.4E+08	Up
1	SCN5A	3.4E+08	Up
1	SCN2B	3.4E+08	Up
1	GRM5	3.4E+08	Up
1	SLC3A2	3.4E+08	Up
1	SYK	3.4E+08	Up
1	SCN9A	3.4E+08	Up
1	CYBA	3.4E+08	Up
1	NOXA1	3.4E+08	Up

Table S4. Top 10 gene associated diseases by DisGeNET

Term	P-value	Adjusted P-value	Odds Ratio	Combined Score
**GSE150316**				
Erythrocyte Mean Corpuscular Hemoglobin Test	0.00273116	0.15148846	12.7950392	75.5294899
Finding of Mean Corpuscular Hemoglobin	0.00273116	0.15148846	12.7950392	75.5294899
Esophageal Diseases	0.00598524	0.15148846	203.857143	1043.4343
Parkinson Disease	0.00676019	0.15148846	6.49875526	32.4723573
Tonsillitis	0.01119492	0.15148846	101.892857	457.732815
Brain Stem Glioma	0.01193707	0.15148846	95.0952381	421.091823
Malignant neoplasm tonsil	0.01267871	0.15148846	89.1473214	389.380441
Squamous cell carcinoma of vulva	0.01341983	0.15148846	83.8991597	361.691127
HER2-positive carcinoma of breast	0.01341983	0.15148846	83.8991597	361.691127
Tonsillar Carcinoma	0.01416042	0.15148846	79.234127	337.323781
**GSE157852**				
Glioblastoma, IDH-Mutant	1.54E-07	2.02E-04	416.104167	6525.96062
Hyperinsulinism	5.88E-06	0.00242433	26.4339623	318.369016
gliosarcoma	1.26E-05	0.00242433	84.4322034	952.757583
Bone Sarcoma	1.47E-05	0.00242433	512.282051	5701.83907
Chromosome 8, trisomy	1.74E-05	0.00242433	75.4507576	827.052502
secondary acute myeloid leukemia	1.89E-05	0.00242433	73.2242647	796.344069
Chronic myeloproliferative disorder	2.18E-05	0.00242433	31.5103668	338.246978
Sarcoidosis	2.29E-05	0.00242433	31.0964187	332.236237
Congenital anomaly of testis	2.32E-05	0.00242433	68.1917808	727.65115
Progressive night blindness	2.32E-05	0.00242433	68.1917808	727.65115
**GSE151879**				
Stomach Carcinoma	9.41E-07	2.55E-04	14.886354	206.572603
Malignant neoplasm of stomach	1.02E-06	2.55E-04	14.744868	203.446305
Malignant neoplasm of lung	1.24E-06	2.55E-04	14.3945857	195.763591
Embryonal Neoplasm	5.43E-06	6.77E-04	113.301136	1373.71351
Colorectal Neoplasms	5.49E-06	6.77E-04	15.5446009	188.298258
Primary malignant neoplasm of lung	8.03E-06	8.25E-04	11.7761293	138.166432
Ganglioglioma	1.14E-05	0.0010033	87.4035088	994.950794
Carcinoma of lung	1.66E-05	0.00128323	10.656326	117.260111
Colorectal Cancer	1.96E-05	0.00134595	10.1600243	110.117387
Glioblastoma	2.62E-05	0.00161683	10.7036159	112.918568
**GSE189706**				
Photosensitivity of skin	3.37E-08	2.71E-05	192.4734	3311.499
Recurrent Staphylococcus aureus infections	3.37E-06	7.21E-04	1249.125	15739.23
Autosomal Recessive Chronic Granulomatous Disease	6.29E-06	7.21E-04	832.6667	9972.499
Perirectal abscess	6.29E-06	7.21E-04	832.6667	9972.499
Liver Abscess	6.29E-06	7.21E-04	832.6667	9972.499
Rectal abscess	6.29E-06	7.21E-04	832.6667	9972.499

Chronic granulomatous disease	6.98E-06	7.21E-04	112.297	1333.291
Obtundation status	8.08E-06	7.21E-04	713.6786	8368.259
Pschomotor retardation	8.08E-06	7.21E-04	713.6786	8368.259
Recurrent bacterial skin infections	1.23E-05	7.63E-04	555.0278	6273.059

Table S5. Prediction of candidate drugs by DSigDB.

Term	P-value	Adjusted P-value	Odds Ratio	Combined Score
**GSE150316**				
CP-690334-01 PC3 UP	2.49E-22	1.08E-19	1151.48077	57280.5598
LY-294002 MCF7 UP	2.55E-21	5.53E-19	545.534247	25868.5106
CP-690334-01 MCF7 UP	5.18E-20	7.49E-18	586.294118	26035.8841
azacyclonol MCF7 UP	2.00E-19	2.17E-17	342.568966	14749.7325
mefloquine MCF7 UP	7.38E-19	6.08E-17	298.526316	12463.4355
prenylamine MCF7 UP	8.52E-19	6.08E-17	294.074074	12235.5081
etoposide HL60 UP	9.81E-19	6.08E-17	289.751825	12014.8318
niclosamide HL60 UP	6.98E-18	3.78E-16	235.916667	9319.63881
terfenadine MCF7 UP	9.79E-18	4.72E-16	227.712644	8918.42033
fendiline MCF7 UP	2.85E-17	1.24E-15	276.069444	10517.4707
**GSE157852**				
9-Methoxyellipticine TTD 00001373	4.75E-06	0.00217096	118.708333	1455.00164
O-Phospho-L-tyrosine BOSS	7.82E-06	0.00217096	99.675	1172.10169
Imatinib mesylate BOSS	9.32E-06	0.00217096	39.3482365	455.785685
PONATINIB CTD 00004976	1.14E-05	0.00217096	87.4035088	994.950794
valsartan CTD 00002971	1.38E-05	0.00217096	81.6557377	913.596388
brefeldin A BOSS	1.81E-05	0.00217096	74.3208955	811.445529
Dasatinib CTD 00004330	2.40E-05	0.00217096	19.565261	208.092368
diphenhydramine BOSS	2.61E-05	0.00217096	65.4901316	691.204621
calcitriol CTD 00005558	2.86E-05	0.00217096	10.5639599	110.527459
Mevastatin TTD 00009287	2.88E-05	0.00217096	341.470085	3570.56067
**GSE151879**				
LUCANTHONE CTD 00006227	2.49E-27	1.54E-24	1384.95	84839.5468
troglitazone CTD 00002415	1.94E-20	6.00E-18	424.54232	19268.8613
testosterone CTD 00006844	7.82E-19	1.61E-16	281280	1.17E+07
etoposide MCF7 DOWN	3.78E-18	5.84E-16	569.857143	22860.9239
piroxicam CTD 00006571	7.01E-17	8.67E-15	144.587361	5378.10185
calcitriol CTD 00005558	6.98E-16	6.61E-14	270615	9444066.51
Enterolactone CTD 00001393	7.49E-16	6.61E-14	128.956204	4491.30856
Phytoestrogens CTD 00007437	2.15E-15	1.66E-13	415.479167	14032.9156
resveratrol CTD 00002483	5.90E-15	4.05E-13	162.190176	5314.10279
vinblastine CTD 00006986	1.31E-14	8.09E-13	135.383562	4327.82107
**GSE189706**				
Lacosamide	1.38E-07	2.04E-05	450.4737	7115.967
riluzole	1.59E-07	2.04E-05	427.9286	6700.134
zonisamide	1.81E-07	2.04E-05	407.5306	6326.449
Dyclonine hydrochloride	2.06E-07	2.04E-05	388.987	5988.956
primidone	3.27E-07	2.59E-05	329.0769	4914.573
amitriptyline	4.43E-07	2.93E-05	294.9901	4315.594
topiramate	1.54E-06	8.73E-05	189.9524	2542.463

Superoxide BOSS	9.49E-05	0.00471	45.63134	422.6605
dioxidanide CTD 00006819	1.48E-04	0.006549	142.5357	1256.481
Tarichatoxin BOSS	2.20E-04	0.008742	115.9709	976.5813
